# Successful Liver Transplantation After Neurologically Determined Death Donation Following Sodium Nitrite Poisoning

**DOI:** 10.7759/cureus.33278

**Published:** 2023-01-02

**Authors:** Umasankar Mathuram Thiyagarajan, A M James Shapiro

**Affiliations:** 1 Surgery, University of Alberta Hospital, Edmonton, CAN

**Keywords:** decompensated liver disease, sodium nitrite poisoining, liver failure, acute poisoning, liver transplantation

## Abstract

Sodium nitrite poisoning has been reported with increasing frequency since 2017 and popularized on social media as an effective means to commit suicide. Though accidental, non-intentional consumption has been reported, it is uncommon. Sodium nitrite is a colorless, odorless, yellowish-white crystalline material that resembles table salt, is easily ingested for self-harm, and is readily accessible through purchase from online portals at low cost. The chemical is used industrially as a curing agent for meat, fish, and cheese, as it inhibits *Clostridium botulinum* and prevents botulism. We herein report a successful case of liver transplantation from an organ donor who suffered brain death after intentional sodium nitrite consumption. Despite conflicting evidence on sodium nitrite's toxic versus protective effects on the liver, our transplant recipient showed normal graft function in the four months following liver transplantation. It would have been helpful and reassuring to have had access to similar positive case reports when deciding to use such a donor.

## Introduction

Sodium nitrite (SN) is a common food preservative and highly soluble in water and alcohol; it is also used medically as an antidote for cyanide poisoning and as an antihypertensive and antioxidant agent [1,2]. Described symptoms vary from tachycardia, hypotension, sweating, and fainting due to methemoglobinemia at lower doses. Oxygen dissociation from hemoglobin is severely inhibited at a high dose causing rapid cyanosis, collapse, coma, and death [2].

In the past 5 years, reports of suicide by sodium nitrite ingestion have escalated considerably [3-5]. The agent is available easily for online purchase with no restrictions; sadly, it is also promoted by online forums as an effective method of inducing suicide [3].

Due to tissue-level severe hypoxia from methemoglobinemia, it is unknown if end organs can recover in acute fatal poisoning [6]. Herein, we successfully transplanted a liver from an SN donor and demonstrated, at least in this case, that the donor liver could recover fully post-transplant and be associated with excellent graft for patients with end-stage liver disease. It would have been helpful and reassuring to have had access to similar case reports when deciding to proceed in this case. An acute shortage of ideal organ donors behoves us to explore the potential of higher risk and extended criteria donors.

## Case presentation

Donor

A 19-year-old female had a history of severe recurrent depression and consumed SN in a suicide attempt. She called the emergency medical services (EMS) immediately following ingestion. She suffered a cardiac arrest secondary to severe methemoglobinemia when EMS arrived at her home. She was resuscitated promptly by EMS and then transferred to the emergency room at the University of Alberta Hospital. On arrival, her blood pressure was 80/46mm Hg, with an oxygen saturation of 60% while receiving 100% FiO2. Treatment was initiated with methylene blue (cumulative dose 7 mg/kg administered intravenously) and plasma exchange. She was also given 4 units of packed RBCs in the emergency to provide her fresh hemoglobin with oxygen-carrying capacity.

She was seen by the cardiovascular intensive care team and underwent an urgent percutaneous venous-arterial extracorporeal membrane oxygenation (ECMO) in the emergency room. Later, she achieved good organ perfusion with satisfactory urine output and declining lactate; however, she progressed to multi-organ failure, as evident in a chest x-ray showing adult respiratory distress syndrome (ARDS). She was declared brain dead 24 hours after without brain stem reflexes and a brain CT scan confirming extensive cerebral edema with brain-stem herniation. Her family was contacted by the organ donation coordinator, and agreed to proceed in line with her wishes. The biochemical profile at the time of donation is shown in table 1; the hematological profile was within normal limits.

**Table 1:** Clinical Laboratory Reference Values

Parameter	Patient’s value	Reference Values
Bilirubin	79 µmol/liter	3 -17 µmol/liter
Alanine aminotransferase (ALT)	95 IU/liter	17- 63 IU/liter
Aspartate aminotransferase (AST)	271 IU/liter	15 - 34 IU/liter
Alkaline Phosphatase (ALP)	98 IU/liter	40 - 120 IU/liter
International normalized ratio (INR)	1.5	0.9 - 1.2
Urea	3 mmol/L	2.1–8.0 mmol/L
Creatinine	79 µmol/L	22–75 µmol/L

Recipient

The recipient was a 68-year-old lady with decompensated liver cirrhosis secondary to primary biliary cirrhosis (PBC). Her decompensation includes intense pruritus, jaundice, esophageal varices, and diuretic-controlled ascites, and her MELDNa (Model for End-stage Liver Disease) score was 15. The biochemical profile showed a bilirubin of 90 µmol/L, ALT 92 IU/L, AST 137 IU/L, albumin 26 gram/L, and INR 1.1; otherwise, renal function and hematological profiles were within normal limits.

Her weight was 72 Kg with a body mass index of 30 at the time of assessment. Her medical history included a hiatus hernia causing gastroesophageal reflux disorder and hypertension. Medications include spironolactone, furosemide, pantoprazole, ramipril, ursodeoxycholic acid, vitamin D, and orbeticholic acid. Surgical history included laparoscopic cholecystectomy and an open appendectomy through a right paramedian incision. She was evaluated for liver transplantation and remained actively listed for transplant for approximately one year.

Liver transplantation and immunosuppression

**Figure 1:**
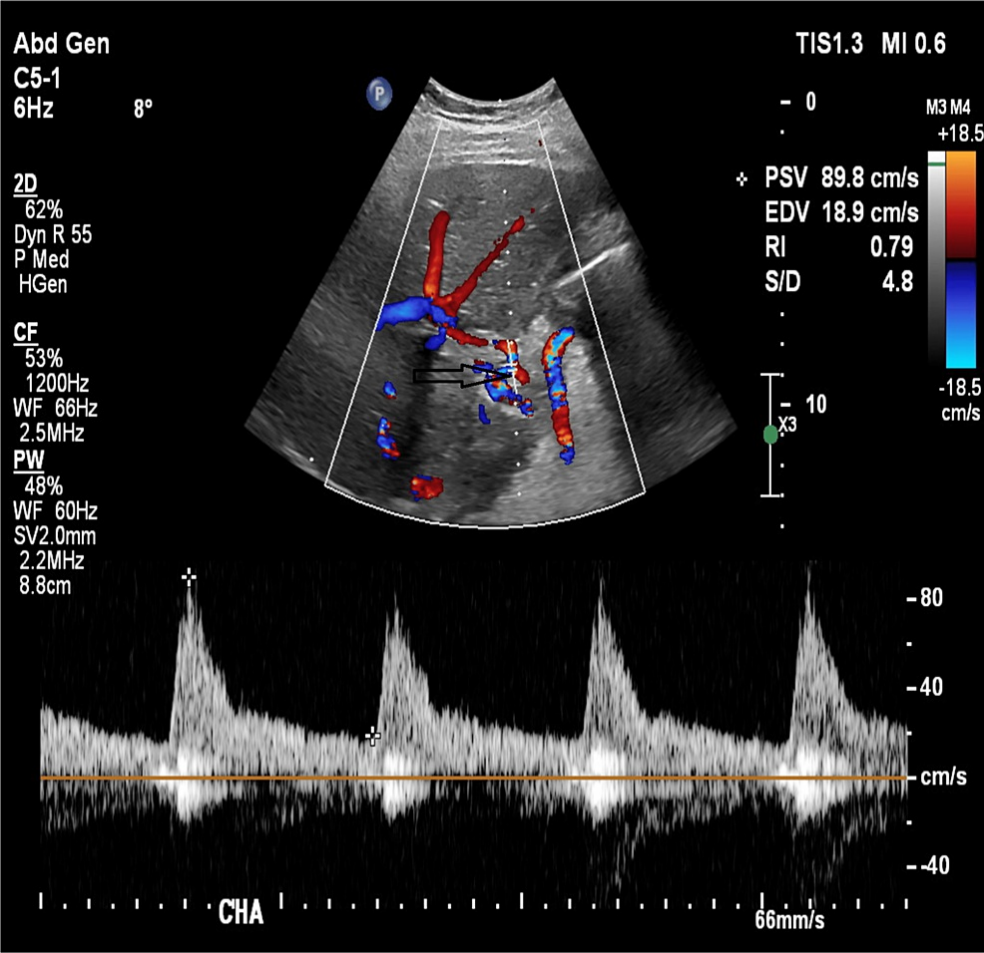
Doppler Examination of the liver on the first postoperative day.

The donor was matched for size and blood group for this recipient, and the liver accepted for transplantation under 'Exceptional Distribution' higher risk after discussion with the recipient. The liver was assessed intraoperatively by the organ retrieval team with a satisfactory macroscopical appearance and no obvious contraindications. The liver transplantation was completed with caval replacement, single artery, and duct-to-duct biliary reconstruction without blood product transfusion. There was no reperfusion injury syndrome, and the recipient was transferred to the intensive care unit and moved to the transplant ward on postoperative day 2. A baseline Doppler liver ultrasound was normal on the first postoperative day, with a satisfactory resistive index at the common hepatic artery (RI-0.79; figure 1).

Immunosuppression consisted of basiliximab induction (Simulect, Novartis Pharmaceuticals Canada Inc, Quebec, Canada) intraoperatively and repeated on day 4; tacrolimus and mycophenolate mofetil, without corticosteroids. Histopathological examination of the explanted liver confirmed primary biliary cirrhosis with no evidence of malignancy.

With satisfactory progress and uneventful recovery from surgery, she was discharged on a postoperative Day 16, with normal liver function (ALT 28) IU/L, AST 29 IU/L, Bilirubin 21 µmol/L, INR 1.1 with normal renal function and hematological profile. The patient was followed as an outpatient regularly, and at four months post liver transplantation enjoys good health with normal liver function.

## Discussion

The impact of SN poisoning on the liver is controversial as the evidence from the literature is conflicting. Studies on mice showed degenerative changes, including increased malondialdehyde and nitrosative tissue damage [7-11]. Moreover, it causes lipid peroxidation, chromosomal aberrations after exposure [8], and the cytoplasmic vacuolization of centrilobular hepatocytes [7]. It has also been shown to induce cytotoxicity from reactive oxygen species formation, mitochondrial injury, and release of lysosomal enzymes leading to cell death [12].

Work by Braunton and Murrell et al. on organic nitrites has demonstrated the potential health benefits of nitrite, including peripheral vasodilatation and cardioprotection which paved the way for nitroglycerine in the treatment of angina pectoris 140 years ago [13,14]. These benefits accrue only at substantially lower drug exposure than acute nitrite poisoning; secondly, inorganic nitrites differ from SN in class, potency, and activity [13,14]. Similar beneficial effects were also shown in hepatic protection in ischemia-reperfusion injury in mice though the mode and concentration are different in the experiments described [15-17].

Though the literature has shown significant liver damage documented in mice, no deleterious effects were noted in humans suffering from acute poisoning. This encouraged us to assess the donor's liver intraoperatively before making a final decision on proceeding with transplantation. As the donor's liver was macroscopically healthy with no concerns or contraindications, we proceeded with donation and liver transplantation.

After 4 months post-transplantation, our choice was correct despite the mild elevation of liver function in the donor. While this single case report is anecdotal, and one cannot assume that SN toxicity is benign in organ donation, it is at least reassuring and may assist other transplant programs in their decision to consider other similar donors. We do not know the ingested dose of SN, and that in itself could be a critical variable in determining the utility and safety of organ donation in other future cases.

## Conclusions

Pressures on liver transplant waiting lists from an acute organ donor shortage often mandate using less-than-ideal donors. A liver derived from a donor with fatal SN poisoning was safely transplanted in this case with an excellent outcome for the recipient. Judicious donor selection is always appropriate to balance risk-benefit, and in SN poisoning, potentially, livers can be transplanted safely in select cases. Given the increasing use and choice of SN as a suicidal agent in the young donor population, other centers are likely to be referred such donors for careful consideration.
